# Challenges in the Diagnosis and Management of Renal Cell Carcinoma With Sarcomatoid Differentiation: A Case Report

**DOI:** 10.7759/cureus.73436

**Published:** 2024-11-11

**Authors:** Evan Ehsan, Ali Z Ansari, Sahar Hafeez, Srihita Patibandla, Samer M Beauti, Nilay Bhatt

**Affiliations:** 1 Department of Emergency Medicine, William Carey University College of Osteopathic Medicine, Hattiesburg, USA; 2 Department of Pathology, William Carey University College of Osteopathic Medicine, Hattiesburg, USA; 3 Department of Internal Medicine, Trinity Health Grand Rapids, Grand Rapids, USA; 4 Department of Internal Medicine, William Carey University College of Osteopathic Medicine, Hattiesburg, USA; 5 Department of Internal Medicine, Merit Health Wesley, Hattiesburg, USA

**Keywords:** abnormal ct, metastasis, right-sided abdominal pain, right-sided pleural effusion, sarcomatoid renal cell carcinoma, shortness of breath (sob), small renal mass, spindle-shaped cells, thoracic radiology, vallecula

## Abstract

Metastatic sarcomatoid renal cell carcinoma (sRCC) is a significant therapeutic and diagnostic challenge due to its rarity and aggressiveness, which contribute to its poor prognosis. This case report presents the case of a 47-year-old Caucasian man with shortness of breath and right-sided abdominal pain. History revealed an extensive smoking history, a left renal mass diagnosed two months ago with inconclusive results, and an enlarged mass on computed tomography (CT) scan one week ago in an emergency department (ED) visit that showed signs consistent with metastatic disease. CT scan on presentation revealed a right posterolateral chest wall mass measuring 8.5×3.5 cm between the 10th and 11th ribs, as well as multiple bilateral pulmonary metastases. CT scan of the head revealed a soft tissue mass anterior to the epiglottis within the vallecula. Left renal pole mass was also consistent with neoplasm, with stable surrounding mass-like densities consistent with adenopathy and involvement of the adrenal gland. Histopathological examination of the 11 cm right chest wall mass biopsy revealed sRCC due to the visualization of spindle to epithelioid tumor with focal clear cell morphology and a prominent vascular network leading to a nested appearance. Management of his symptoms included thoracocentesis of the pleural effusion, nasal cannula due to low partial pressure of oxygen (PO2), pleurodesis, and trending down of the hypercalcemia. Oncology confirmed the spindle cell neoplasm due to stage IV renal cancer; the patient was transferred to hematology-oncology for further evaluation, but soon after succumbed to the complexities of metastatic disease. This case highlights the challenges of the management and diagnosis of metastatic sRCC over a three-week inpatient service, ultimately revealing a poor prognosis due to the aggressiveness and time of diagnosis, emphasizing the need for early detection methods and personalized treatment strategies. Further research is needed to explore novel therapeutic approaches to tackle this rare and aggressive variant of RCC.

## Introduction

Renal cell carcinoma (RCC) is the most common type of cancer arising from the kidney, making up more than 90% of all cases [1]. It is also the 13th most common malignancy worldwide with 400,000 new cases and 175,000 deaths yearly [2]. RCC can be broken up into subtypes, diagnosed by imaging, including clear cell, papillary, and chromophobe histological subtypes, with clear cell morphologies making up a majority of RCCs [3]. Appearing in only 5% of all RCCs [1], metastatic sarcomatoid RCC (sRCC) is a rare and aggressive variant of renal cancers, characterized by its histopathological features and presence in any subtype of RCC. With RCC having a one in four chance to have already metastasized by the time of diagnosis, sRCC is no different in its propensity for early metastasis, with 75% of all sRCCs metastasizing leading to poor outcomes [4].

sRCC shares similar risk factors as RCC, with smoking being the biggest risk factor. Studies show a strong dose-dependent increase in risk associated with the number of cigarettes smoked per day and a substantial reduction in risk for long-term former smokers [5]. sRCC also commonly appears in ages 50-70, affecting men more than women (2:1 ratio) [6].

Clinically, metastatic sRCC most commonly travels to the lungs (>50%), bone (33%), lymphatic nodes, liver, and brain, respectively [7]. With sarcomatoid tumors typically being large (9-10 cm), patients are mostly symptomatic with symptoms correlating to sites of metastases. Fine needle aspiration or standard core biopsy is typically required to diagnose sRCC [8]. Histologically, sRCC presents as a transformative growth pattern of the epithelial neoplasm into spindle-shaped cells, containing both epithelial (carcinomatous) and mesenchymal (sarcomatoid) components, unlike a true sarcoma of the kidney [2].

In this case report, we discuss the complexities of the management and diagnosis of metastatic sarcomatoid renal cell carcinoma in a 47-year-old man who presents with shortness of breath and abdominal pain. As we follow this patient, we discuss symptomatic relief, clinical procedures, and lab findings that help diagnose metastatic sRCC. With poor median overall survival of 5-12 months [4], we discuss the limited treatment options and emphasize the importance of novel therapeutic methods.

## Case presentation

A 47-year-old Caucasian man presents to the emergency department (ED) with severe right-sided abdominal pain and shortness of breath. The patient reported that the abdominal pain had started over a week ago and had progressively worsened. He has a past medical history of appendectomy, cerebral vascular accident (CVA), heart attack, and tonsillectomy. Notably, a left renal mass was incidentally discovered during his workup for his appendectomy two months ago, with a biopsy of the mass showing inconclusive results. A computed tomography (CT) scan performed one week prior to his ED visit revealed enlargement of the left renal mass with signs of metastatic disease, with multiple soft tissues in the fat. The patient reported worsening right-sided abdominal pain over the past two months, worsening shortness of breath during the past week, and occasional cough with brown sputum. He had a significant smoking history, smoking one pack per day recently and at one point smoking four packs per day for the past 39 years.

Physical examination showed a large abdominal mass on the right side, moderate tenderness on palpation, diminished breath sounds on the right lung base, and tachycardia with a normal rhythm. Bowel sounds were present and normoactive in all four quadrants. Initially, the patient appeared well and in no acute distress with vitals revealing saturation of peripheral oxygen (SpO2) decreased at 92% on room air, a heart rate of 118 beats per minute (bpm), a temperature of 98°F (36.7°C), a respiration rate of 20 breaths per minute (bpm), a blood pressure of 141/85 mmHg, and a body mass index (BMI) of 36.4.

**Figure 1:**
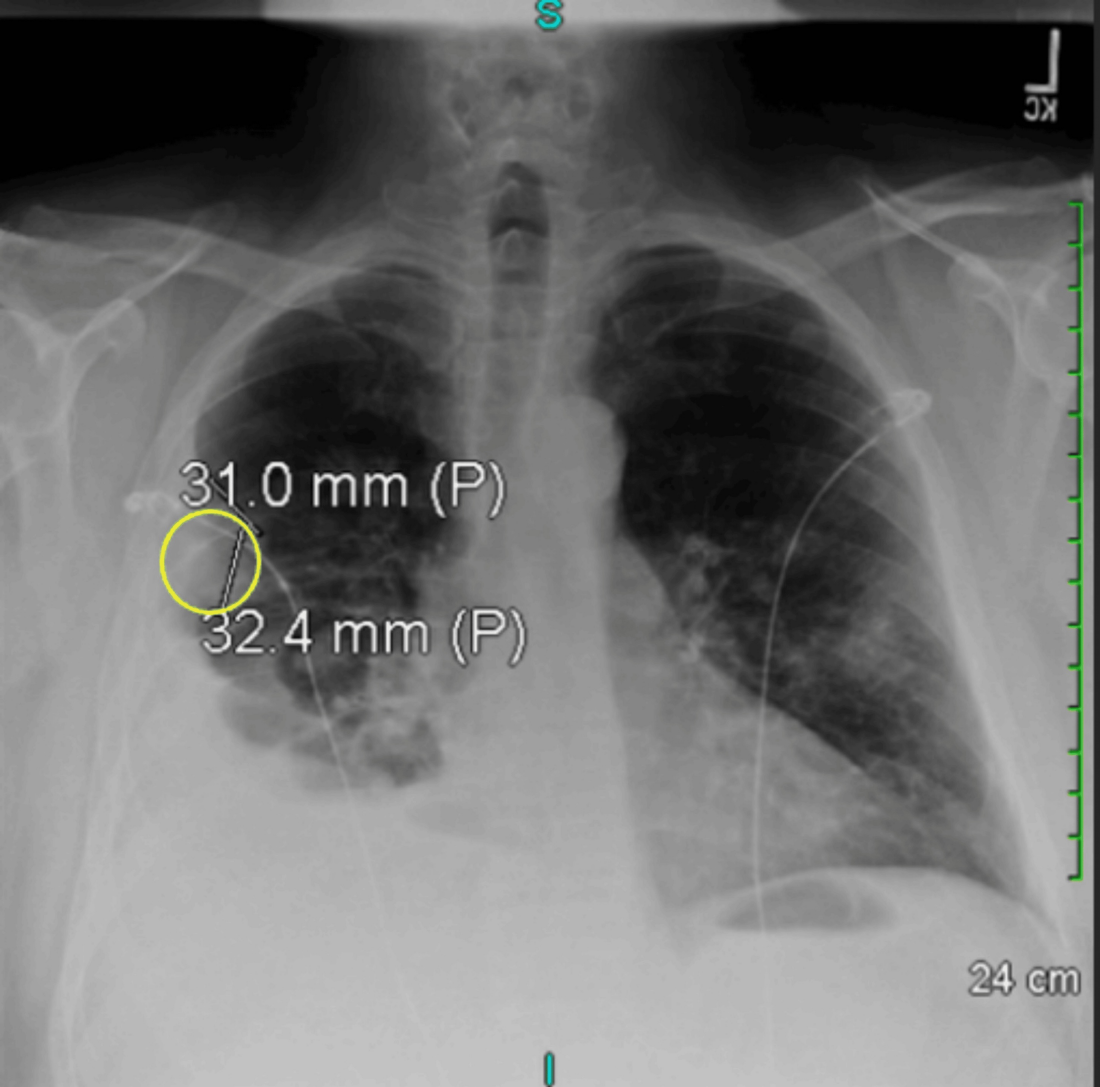
Initial radiologic imaging showing moderate right pleural effusion seen as well as multiple bilateral soft tissue nodules scattered throughout. The largest nodule seen is pleural-based on the right measuring approximately 31×32.4 mm in the right upper lobe (yellow circle).

**Figure 2:**
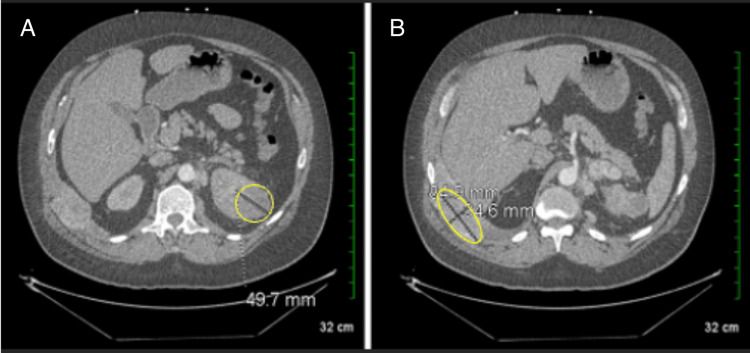
(A) CTA showing a left upper pole renal mass measuring 49.7 mm (yellow circle), with stable surrounding mass-like densities indicative of adenopathy. (B) Imaging of the right chest wall revealing a mass between the 10th and 11th ribs, measuring 84.9×34.6 mm (yellow circle), accompanied by destructive changes in the right 11th rib and a fracture of the right 10th rib. CTA: computed tomography angiography

An initial chest X-ray was performed, revealing a moderate right pleural effusion and multiple bilateral soft tissue nodules suspicious of metastatic disease (Figure 1). To rule out pulmonary embolism, a coronary CT angiography (CTA) of the chest was performed, confirming metastatic disease with multiple pleural-based masses and pulmonary nodules. There was a right posterolateral chest wall mass measuring up to a maximum of about 8.5×3.5 cm in size between the right 10th and 11th ribs, with destructive changes involving the 11th rib and a fracture of the adjacent 10th rib laterally (Figure 2).

Laboratory investigations were ordered, including a complete blood count (CBC) and comprehensive metabolic panel (CMP). The CBC and CMP revealed mild anemia, hypercalcemia, hyponatremia, thrombocytosis, leukocytosis, elevated blood urea nitrogen (BUN), and creatinine (Cr) with a BUN/Cr ratio of 12.1 (Table 1).

**Table 1:** Laboratory findings indicating leukocytosis, anemia, thrombocytosis, hyponatremia, hypercalcemia, and elevated levels of blood urea nitrogen and creatinine.

Test	Observed value	Reference range
White blood cells	12.8×10³/μL	4-11×10³/μL
Hemoglobin	11.9 g/dL	12.1-15.1 g/dL
Hematocrit	36.8%	42-52%
Platelet count	572×10⁹/L	150-450×10⁹/L
Sodium	134 mmol/L	135-147 mmol/L
Potassium	4.1 mmol/L	3.5-5 mmol/L
Chloride	97 mmol/L	96-106 mmol/L
Calcium	11.9 mg/dL	8.4-10.2 mg/dL
Carbon dioxide	28 mmol/L	23-29 mmol/L
Blood urea nitrogen	15 mmol/L	2.1-8.5 mmol/L
Creatinine	1.24 mg/dL	0.6-1.2 mg/dL

Given the concern for malignancy versus infection, the patient was admitted for further evaluation and management. Despite intravenous (IV) fluid administration for hypercalcemia and pain control, the patient's symptoms persisted. Although calcium and liver enzymes started trending down, suspicion of hepatic malignancy remained. A thoracocentesis of the pleural effusion revealed no malignant cells, some mesothelial cells with reactive changes, few lymphocytes, and occasional neutrophils. 

Further imaging, crucial in the initial detection, characterization, and staging of renal masses suspected of sRCC, was performed. A CT scan of the abdomen and pelvis revealed soft tissue masses over the right posterior costophrenic angle with some rib destruction in this area, a left kidney mass, a left adrenal mass, and multiple soft tissue masses in the fat of the left upper quadrant. The liver, spleen, pancreas, and gallbladder were normal.

**Figure 3:**
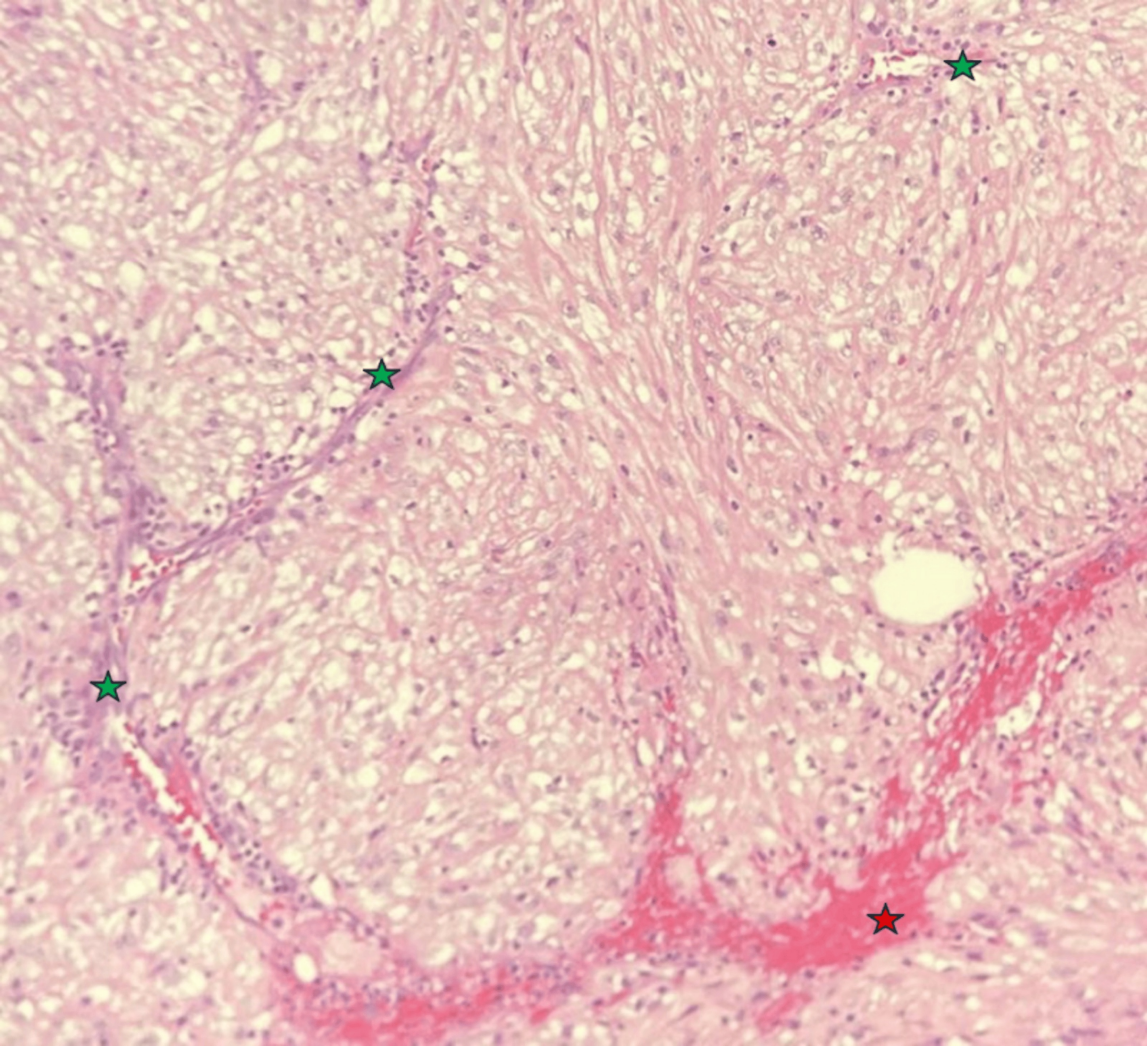
Histopathology photomicrograph I (200× magnification) Nests of cells with clear cytoplasm and small nuclei are separated by delicate septae containing thin-walled blood vessels (indicated by green stars). An area of necrosis (indicated by a red star) is surrounded by cells with eosinophilic cytoplasm.

**Figure 4:**
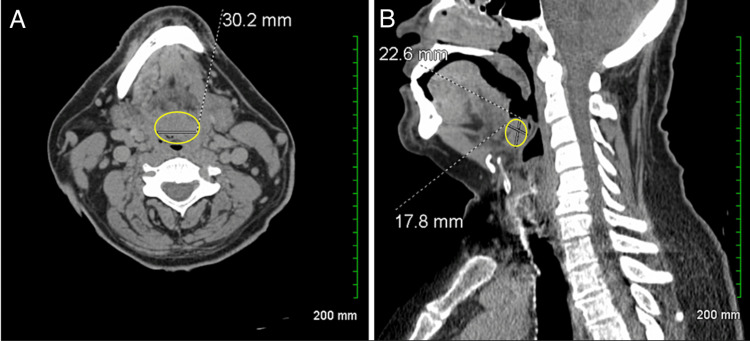
(A) CT scan revealing a 22.6×17.8×30.2 mm soft tissue mass (yellow circles) located just anterior to the epiglottis within the vallecula. (B) Also noted are a few scattered small reactive lymph nodes within the posterior cervical triangle, as well as mild subglottic airway narrowing. CT: computed tomography

Percutaneous CT scan-guided needle biopsy of the pleural-based mass was performed, and microscopic examination confirmed metastatic sRCC, supported by the appearance of a spindle to epithelioid tumor with focal clear cell morphology and prominent vascular network with nested appearance (Figure 3). Submitted immunohistochemical stains were reviewed and show that the tumor cells in the biopsy specimen are positive for CK8/18 while negative for AE1/3, CD34, high molecular weight cytokeratin, CK20, RCC, desmin, myosin, smooth muscle actin (SMA), S100, Melan A, cytokeratin 7 (CK7), and GATA3. Due to the possibility of spindle cell cancer, a CT scan of the neck was performed and revealed a 2.3×1.8×3 cm soft tissue mass anterior to the epiglottis within the vallecula, raising concern for metastatic disease to the head and neck region (Figure 4). Ear, nose, and throat (ENT) was consulted.

Pleural effusions reaccumulated fairly quickly and were drained via a PleurX catheter, and pleurodesis was indicated. A video-assisted thoracoscopic surgery with pleurodesis and biopsy of pleural studding on the right side was obtained with no complications. The biopsy was analyzed, and the metastatic sRCC was confirmed. Oncology consultation categorized the carcinoma as stage IV renal cancer, and the patient chose to proceed with treatment. Shortly after treatment, the patient ultimately succumbed to his disease.

## Discussion

Metastatic sRCC represents a rare and aggressive variant of RCCs, characterized by a propensity for early metastases and distinct histological features. This case displaying a 47-year-old man presenting with abdominal pain and shortness of breath underlines several key features regarding the diagnosis, management, and outcome of this challenging malignancy.

**Figure 5:**
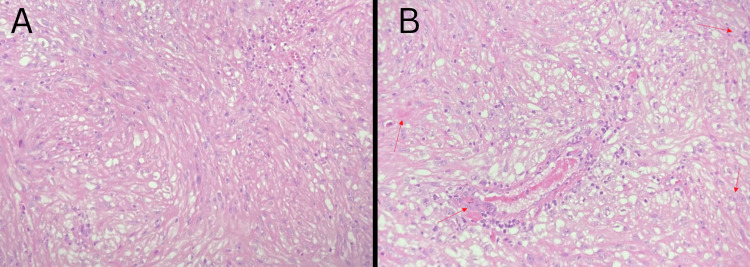
Histopathology photomicrograph II Left: Biopsy specimen showing an admixture of polygonal cells with clear cytoplasm and spindle-shaped cells (a distinct feature of clear cell RCC with sarcomatoid differentiation). Right: Biopsy specimen in high power (40×) showing an intra-tumor cyst surrounded by multiple cells with eosinophilic cytoplasm and giant cells (red arrow). RCC: renal cell carcinoma

Histologically, sRCC presents as a transformative growth pattern of the epithelial neoplasm into spindle-shaped cells, with focal clear cell morphology and a prominent vascular network with a nested appearance. The presence of staghorn blood vessels, nucleolar prominence, and some multinucleated giant cells indicate malignancy (Figure 5). A CK7 immunostain was negative. A positive reticulin and Ki67 stain indicate a form of higher malignancy [4]. A positive vimentin stain along with the proliferation of spindle cells indicates a mesenchymal origin of the malignancy [9]. Typically, an RCC immunostain can indicate certain types of RCC; however, sRCC stains poorly due to the RCC immunostain being epithelial in origin. This histological variant is associated with high aggressiveness and early metastases to distant sites, including the lungs, bone, liver, nodes, and brain. In this case, the patient presented with abdominal pain and shortness of breath due to the sRCC metastasizing to the lungs, with a right upper lobe mass pushing into the abdomen.

Diagnosing sarcomatoid renal cell carcinoma involves a multifaceted approach combining clinical assessment, advanced imaging, and definitive histopathological analysis. The present challenges are due to its rarity and various presentations. sRCC areas may be heterogeneous; a biopsy might miss sarcomatoid components if not sampled adequately. Symptoms are often non-specific and may correlate to its site of metastases, but can present as many other differential diagnoses such as primary renal sarcoma, lymphoma, angiomyolipoma, other variants of renal cell carcinoma, metastatic urothelial carcinoma, and metastatic lung carcinoma due to smoking history and presentation. Oftentimes, the telltale signs of malignant RCC, such as hematuria, are the last symptoms to present [6]. The diagnosis was confirmed with a CT scan-guided needle biopsy of the right-sided pleural-based mass, which revealed the spindle cells with focal clear cell morphology, consistent with metastatic sRCC. However, the initial biopsy of the left upper pole renal mass was inconclusive, highlighting the difficulty in obtaining the definitive diagnosis earlier.

The management of metastatic sRCC has its challenges and often includes a multidisciplinary approach. Treatment options may include surgery, systemic therapy, radiation therapy, and supportive care. Systemic targeted molecular therapies have been replaced by vascular endothelial growth factor tyrosine kinase inhibitors (VEGF-TKIs) such as sunitinib or sorafenib, followed by partial or total nephrectomy with immunotherapy in advanced stages of sRCC. However, the prognosis remains poor as even with treatment, the overall median survival is under a year [6]. Current biomarker studies show that programmed cell death marker 1 (PD-1) and its ligand (PD-L1) were expressed at a much higher level in sRCC than non-sRCC. PD-1 inhibitors such as nivolumab combined with ipilimumab have shown a median overall survival rate of 31 months compared to the VEGF-targeted monotherapy group with sunitinib (14 months) [4]. These findings show potential shifts in the treatment paradigm and shifting therapeutic decisions towards checkpoint inhibitors for sRCC, underlying a desperate need for further investigation into the mechanisms and treatment methods to further prolong median overall survival.

In this case, the patient underwent thoracocentesis for symptomatic relief of the pleural effusions, IV fluid administration for hypercalcemia, and pain management. Given the bilateral pulmonary nodules, right pleural mass, and vallecular mass, the patient's prognosis was poor. The patient opted into radiation therapy, but soon after ultimately succumbed to his disease, underlying the aggressive nature of metastatic sRCC and the limited efficacy of current therapeutic modalities.

The prognosis of metastatic sRCC is relatively poor, with an overall median survival ranging from five to 12 months. Early diagnosis and personalized treatment strategies are desperately needed to optimize patient outcomes. Early detection methods prove difficult with renal core biopsies being inconclusive, due to <50% of the tumor presenting with sarcomatoid features. With percutaneous biopsies from large heterogeneous masses being inconclusive and error-prone, a multi-quadrant technique has shown significant efficacy in spotting sarcomatoid features, with a rate of 86.7% compared to the standard biopsy technique at 25%. Detection in stage I or II sRCC with subsequent early nephrectomy has shown effectiveness in the median overall survival for these patients [10]. While not routinely required for diagnosis, ongoing research exploring molecular alterations associated with sRCC is needed, which may lead to prognostic or therapeutic implications.

Prevention of metastatic sRCC emphasizes cessation of smoking, with a substantial reduction in risk for long-term former smokers [5]. While specific risk factors for sRCC have yet to be shown, obesity and hypertension remain established risk factors for kidney cancers. Maintaining a healthy weight and avoiding smoking have been shown to be effective in reducing the risk of developing renal cancers [11]. Moreover, psychosocial support can help patients and families cope with the emotional and practical challenges when dealing with metastatic sRCC.

## Conclusions

Metastatic sRCC presents as a rather challenging disease characterized by its aggressive behavior, diagnostic complexity, and limited treatment with poor prognosis, making early detection and accurate diagnosis crucial for the optimal management and improvement of patient outcomes. Despite advances in diagnostic imaging and histopathological methods, confirmation of the malignancy can be tricky due to its rare nature and histological heterogeneity. The management of sRCC remains multidisciplinary, involving surgery, systemic therapy, radiation therapy, symptomatic management, and supportive measures. With the average survival time being only a few months, the prognosis for these individuals is unfortunate. In the case presented, despite aggressive treatment soon after definitive diagnosis, the patient succumbed to his illness, highlighting the urgent need for novel therapeutic strategies. Further research is warranted to explore the molecular mechanisms, better methods for early detection, and targeted therapies specifically for sRCC, to improve patient outcomes in this challenging clinical scenario.

## References

[1] Blum KA, Gupta S, Tickoo SK (2020). Sarcomatoid renal cell carcinoma: biology, natural history and management. Nat Rev Urol.

[2] Donskov F (2021). Renal cell carcinoma with non-clear cell histology or sarcomatoid differentiation: recent insight in an unmet clinical need. Ann Transl Med.

[3] Muglia VF, Prando A (2015). Renal cell carcinoma: histological classification and correlation with imaging findings. Radiol Bras.

[4] Pichler R, Compérat E, Klatte T, Pichler M, Loidl W, Lusuardi L, Schmidinger M (2019). Renal cell carcinoma with sarcomatoid features: finally new therapeutic hope?. Cancers (Basel).

[5] Hunt JD, van der Hel OL, McMillan GP, Boffetta P, Brennan P (2005). Renal cell carcinoma in relation to cigarette smoking: meta-analysis of 24 studies. Int J Cancer.

[6] Siegel RL, Miller KD, Jemal A (2020). Cancer statistics, 2020. CA Cancer J Clin.

[7] Cangiano T, Liao J, Naitoh J, Dorey F, Figlin R, Belldegrun A (1999). Sarcomatoid renal cell carcinoma: biologic behavior, prognosis, and response to combined surgical resection and immunotherapy. J Clin Oncol.

[8] Shuch B, Bratslavsky G, Linehan WM, Srinivasan R (2012). Sarcomatoid renal cell carcinoma: a comprehensive review of the biology and current treatment strategies. Oncologist.

[9] Zhang H, Majeed NK, Sharifi R, Guzman G (2019). A case of sarcomatoid renal cell carcinoma with osseous metaplasia and papillary renal cell carcinoma metastasis. Clin Pathol.

[10] Abel EJ, Heckman JE, Hinshaw L (2015). Multi-quadrant biopsy technique improves diagnostic ability in large heterogeneous renal masses. J Urol.

[11] Tahbaz R, Schmid M, Merseburger AS (2018). Prevention of kidney cancer incidence and recurrence: lifestyle, medication and nutrition. Curr Opin Urol.

